# Supplementary material for: Pyruvate Dehydrogenase Kinase 1 inhibition mediated oxidative phosphorylation enhancement in cartilage promotes osteoarthritis progression
Source: BMC Musculoskelet Disord. 2023 Jul 20;24:597. doi: 10.1186/s12891-023-06585-6 (PMC10357736; doi:10.1186/s12891-023-06585-6)
Supplement: Supplementary file 1 — Additional file 1. [file 12891_2023_6585_MOESM1_ESM.zip › BP.Gsea.1653623667859/GOBP_ENDOTHELIUM_DEVELOPMENT.html]

Details for gene set GOBP\_ENDOTHELIUM\_DEVELOPMENT[GSEA]

|  || Dataset | 55457\_collapsed\_to\_symbols.cls.cls #OA\_versus\_normal.cls.cls #OA\_versus\_normal\_repos |
| Phenotype | cls.cls#OA\_versus\_normal\_repos |
| Upregulated in class | normal |
| GeneSet | GOBP\_ENDOTHELIUM\_DEVELOPMENT |
| Enrichment Score (ES) | -0.4152174 |
| Normalized Enrichment Score (NES) | -1.8642365 |
| Nominal p-value | 0.0 |
| FDR q-value | 0.06281561 |
| FWER p-Value | 0.849 |
Table: GSEA Results Summary

  

Fig 1: Enrichment plot: GOBP\_ENDOTHELIUM\_DEVELOPMENT      
 Profile of the Running ES Score & Positions of GeneSet Members on the Rank Ordered List

  

| SYMBOL | TITLE | RANK IN GENE LIST | RANK METRIC SCORE | RUNNING ES | CORE ENRICHMENT || 1 | TMEM100 | transmembrane protein 100 [Source:HGNC Symbol;Acc:HGNC:25607] | 389 | 0.710 | -0.0108 | No |
| 2 | RAP2C | "RAP2C, member of RAS oncogene family [Source:HGNC Symbol;Acc:HGNC:21165]" | 404 | 0.705 | 0.0075 | No |
| 3 | GSTM3 | glutathione S-transferase mu 3 [Source:HGNC Symbol;Acc:HGNC:4635] | 1025 | 0.512 | -0.0268 | No |
| 4 | EDF1 | endothelial differentiation related factor 1 [Source:HGNC Symbol;Acc:HGNC:3164] | 1684 | 0.401 | -0.0672 | No |
| 5 | CXCL10 | C-X-C motif chemokine ligand 10 [Source:HGNC Symbol;Acc:HGNC:10637] | 1709 | 0.396 | -0.0581 | No |
| 6 | CSNK2B | casein kinase 2 beta [Source:HGNC Symbol;Acc:HGNC:2460] | 1788 | 0.385 | -0.0536 | No |
| 7 | TNF | tumor necrosis factor [Source:HGNC Symbol;Acc:HGNC:11892] | 2098 | 0.350 | -0.0681 | No |
| 8 | HPSE | heparanase [Source:HGNC Symbol;Acc:HGNC:5164] | 2360 | 0.319 | -0.0797 | No |
| 9 | RAB1B | "RAB1B, member RAS oncogene family [Source:HGNC Symbol;Acc:HGNC:18370]" | 2453 | 0.308 | -0.0784 | No |
| 10 | CLDN1 | claudin 1 [Source:HGNC Symbol;Acc:HGNC:2032] | 3089 | 0.241 | -0.1214 | No |
| 11 | HOXB5 | homeobox B5 [Source:HGNC Symbol;Acc:HGNC:5116] | 3326 | 0.219 | -0.1338 | No |
| 12 | ID1 | "inhibitor of DNA binding 1, HLH protein [Source:HGNC Symbol;Acc:HGNC:5360]" | 3349 | 0.216 | -0.1295 | No |
| 13 | PLOD3 | "procollagen-lysine,2-oxoglutarate 5-dioxygenase 3 [Source:HGNC Symbol;Acc:HGNC:9083]" | 3614 | 0.192 | -0.1449 | No |
| 14 | NRP1 | neuropilin 1 [Source:HGNC Symbol;Acc:HGNC:8004] | 3704 | 0.185 | -0.1467 | No |
| 15 | PLCB1 | phospholipase C beta 1 [Source:HGNC Symbol;Acc:HGNC:15917] | 3724 | 0.183 | -0.1432 | No |
| 16 | EZR | ezrin [Source:HGNC Symbol;Acc:HGNC:12691] | 3817 | 0.174 | -0.1455 | No |
| 17 | ADD1 | adducin 1 [Source:HGNC Symbol;Acc:HGNC:243] | 3872 | 0.167 | -0.1452 | No |
| 18 | ACVR1 | activin A receptor type 1 [Source:HGNC Symbol;Acc:HGNC:171] | 3898 | 0.165 | -0.1426 | No |
| 19 | HEY2 | hes related family bHLH transcription factor with YRPW motif 2 [Source:HGNC Symbol;Acc:HGNC:4881] | 4058 | 0.150 | -0.1508 | No |
| 20 | HEG1 | heart development protein with EGF like domains 1 [Source:HGNC Symbol;Acc:HGNC:29227] | 4117 | 0.144 | -0.1514 | No |
| 21 | PROX1 | prospero homeobox 1 [Source:HGNC Symbol;Acc:HGNC:9459] | 4299 | 0.128 | -0.1620 | No |
| 22 | CD34 | CD34 molecule [Source:HGNC Symbol;Acc:HGNC:1662] | 4341 | 0.125 | -0.1618 | No |
| 23 | FGF1 | fibroblast growth factor 1 [Source:HGNC Symbol;Acc:HGNC:3665] | 4436 | 0.118 | -0.1659 | No |
| 24 | EDNRA | endothelin receptor type A [Source:HGNC Symbol;Acc:HGNC:3179] | 4663 | 0.099 | -0.1808 | No |
| 25 | HAPLN2 | hyaluronan and proteoglycan link protein 2 [Source:HGNC Symbol;Acc:HGNC:17410] | 4834 | 0.084 | -0.1917 | No |
| 26 | PDPN | podoplanin [Source:HGNC Symbol;Acc:HGNC:29602] | 5594 | 0.024 | -0.2504 | No |
| 27 | IKBKB | inhibitor of nuclear factor kappa B kinase subunit beta [Source:HGNC Symbol;Acc:HGNC:5960] | 5914 | 0.001 | -0.2753 | No |
| 28 | SOX18 | SRY-box transcription factor 18 [Source:HGNC Symbol;Acc:HGNC:11194] | 6053 | -0.008 | -0.2859 | No |
| 29 | PPP1R16B | protein phosphatase 1 regulatory subunit 16B [Source:HGNC Symbol;Acc:HGNC:15850] | 6283 | -0.026 | -0.3031 | No |
| 30 | CXCR4 | C-X-C motif chemokine receptor 4 [Source:HGNC Symbol;Acc:HGNC:2561] | 6382 | -0.034 | -0.3098 | No |
| 31 | FOXJ2 | forkhead box J2 [Source:HGNC Symbol;Acc:HGNC:24818] | 6411 | -0.036 | -0.3110 | No |
| 32 | GJA4 | gap junction protein alpha 4 [Source:HGNC Symbol;Acc:HGNC:4278] | 6548 | -0.045 | -0.3204 | No |
| 33 | RAP1A | "RAP1A, member of RAS oncogene family [Source:HGNC Symbol;Acc:HGNC:9855]" | 6580 | -0.048 | -0.3215 | No |
| 34 | MYD88 | MYD88 innate immune signal transduction adaptor [Source:HGNC Symbol;Acc:HGNC:7562] | 6638 | -0.052 | -0.3245 | No |
| 35 | RAP2B | "RAP2B, member of RAS oncogene family [Source:HGNC Symbol;Acc:HGNC:9862]" | 6749 | -0.059 | -0.3315 | No |
| 36 | MSN | moesin [Source:HGNC Symbol;Acc:HGNC:7373] | 6919 | -0.074 | -0.3426 | No |
| 37 | CEACAM1 | CEA cell adhesion molecule 1 [Source:HGNC Symbol;Acc:HGNC:1814] | 6960 | -0.077 | -0.3436 | No |
| 38 | RAP1B | "RAP1B, member of RAS oncogene family [Source:HGNC Symbol;Acc:HGNC:9857]" | 6980 | -0.078 | -0.3430 | No |
| 39 | PRKD2 | protein kinase D2 [Source:HGNC Symbol;Acc:HGNC:17293] | 7011 | -0.081 | -0.3431 | No |
| 40 | FOXP3 | forkhead box P3 [Source:HGNC Symbol;Acc:HGNC:6106] | 7035 | -0.082 | -0.3426 | No |
| 41 | AFDN | "afadin, adherens junction formation factor [Source:HGNC Symbol;Acc:HGNC:7137]" | 7077 | -0.086 | -0.3434 | No |
| 42 | MET | "MET proto-oncogene, receptor tyrosine kinase [Source:HGNC Symbol;Acc:HGNC:7029]" | 7322 | -0.104 | -0.3597 | No |
| 43 | ETV2 | ETS variant transcription factor 2 [Source:HGNC Symbol;Acc:HGNC:3491] | 7365 | -0.108 | -0.3600 | No |
| 44 | VCL | vinculin [Source:HGNC Symbol;Acc:HGNC:12665] | 7687 | -0.133 | -0.3814 | No |
| 45 | PROC | "protein C, inactivator of coagulation factors Va and VIIIa [Source:HGNC Symbol;Acc:HGNC:9451]" | 7786 | -0.140 | -0.3852 | No |
| 46 | BMP4 | bone morphogenetic protein 4 [Source:HGNC Symbol;Acc:HGNC:1071] | 7816 | -0.142 | -0.3835 | No |
| 47 | ZEB1 | zinc finger E-box binding homeobox 1 [Source:HGNC Symbol;Acc:HGNC:11642] | 8147 | -0.166 | -0.4047 | No |
| 48 | S1PR2 | sphingosine-1-phosphate receptor 2 [Source:HGNC Symbol;Acc:HGNC:3169] | 8154 | -0.167 | -0.4006 | No |
| 49 | TNFRSF1A | TNF receptor superfamily member 1A [Source:HGNC Symbol;Acc:HGNC:11916] | 8196 | -0.171 | -0.3991 | No |
| 50 | HEY1 | hes related family bHLH transcription factor with YRPW motif 1 [Source:HGNC Symbol;Acc:HGNC:4880] | 8213 | -0.172 | -0.3956 | No |
| 51 | XDH | xanthine dehydrogenase [Source:HGNC Symbol;Acc:HGNC:12805] | 8366 | -0.183 | -0.4024 | No |
| 52 | TJP1 | tight junction protein 1 [Source:HGNC Symbol;Acc:HGNC:11827] | 8523 | -0.195 | -0.4092 | No |
| 53 | RHOA | ras homolog family member A [Source:HGNC Symbol;Acc:HGNC:667] | 8601 | -0.201 | -0.4097 | Yes |
| 54 | VEZF1 | vascular endothelial zinc finger 1 [Source:HGNC Symbol;Acc:HGNC:12949] | 8621 | -0.202 | -0.4056 | Yes |
| 55 | F2RL1 | F2R like trypsin receptor 1 [Source:HGNC Symbol;Acc:HGNC:3538] | 8631 | -0.203 | -0.4007 | Yes |
| 56 | PDE4D | phosphodiesterase 4D [Source:HGNC Symbol;Acc:HGNC:8783] | 8797 | -0.216 | -0.4076 | Yes |
| 57 | RBPJ | recombination signal binding protein for immunoglobulin kappa J region [Source:HGNC Symbol;Acc:HGNC:5724] | 8800 | -0.217 | -0.4018 | Yes |
| 58 | PDE2A | phosphodiesterase 2A [Source:HGNC Symbol;Acc:HGNC:8777] | 8872 | -0.223 | -0.4012 | Yes |
| 59 | COL15A1 | collagen type XV alpha 1 chain [Source:HGNC Symbol;Acc:HGNC:2192] | 8954 | -0.228 | -0.4012 | Yes |
| 60 | RAPGEF6 | Rap guanine nucleotide exchange factor 6 [Source:HGNC Symbol;Acc:HGNC:20655] | 8993 | -0.231 | -0.3978 | Yes |
| 61 | COL18A1 | collagen type XVIII alpha 1 chain [Source:HGNC Symbol;Acc:HGNC:2195] | 9003 | -0.232 | -0.3921 | Yes |
| 62 | SMAD4 | SMAD family member 4 [Source:HGNC Symbol;Acc:HGNC:6770] | 9035 | -0.235 | -0.3881 | Yes |
| 63 | NRG1 | neuregulin 1 [Source:HGNC Symbol;Acc:HGNC:7997] | 9209 | -0.248 | -0.3948 | Yes |
| 64 | GDF2 | growth differentiation factor 2 [Source:HGNC Symbol;Acc:HGNC:4217] | 9223 | -0.250 | -0.3889 | Yes |
| 65 | RDX | radixin [Source:HGNC Symbol;Acc:HGNC:9944] | 9239 | -0.251 | -0.3831 | Yes |
| 66 | ADAMTS12 | ADAM metallopeptidase with thrombospondin type 1 motif 12 [Source:HGNC Symbol;Acc:HGNC:14605] | 9360 | -0.260 | -0.3853 | Yes |
| 67 | CLIC4 | chloride intracellular channel 4 [Source:HGNC Symbol;Acc:HGNC:13518] | 9537 | -0.277 | -0.3914 | Yes |
| 68 | TIE1 | tyrosine kinase with immunoglobulin like and EGF like domains 1 [Source:HGNC Symbol;Acc:HGNC:11809] | 9548 | -0.278 | -0.3845 | Yes |
| 69 | RAB1A | "RAB1A, member RAS oncogene family [Source:HGNC Symbol;Acc:HGNC:9758]" | 9604 | -0.284 | -0.3810 | Yes |
| 70 | ICAM1 | intercellular adhesion molecule 1 [Source:HGNC Symbol;Acc:HGNC:5344] | 9740 | -0.296 | -0.3834 | Yes |
| 71 | F11R | F11 receptor [Source:HGNC Symbol;Acc:HGNC:14685] | 9765 | -0.297 | -0.3771 | Yes |
| 72 | TJP2 | tight junction protein 2 [Source:HGNC Symbol;Acc:HGNC:11828] | 9772 | -0.298 | -0.3693 | Yes |
| 73 | CLDN5 | claudin 5 [Source:HGNC Symbol;Acc:HGNC:2047] | 9816 | -0.302 | -0.3643 | Yes |
| 74 | CDH5 | cadherin 5 [Source:HGNC Symbol;Acc:HGNC:1764] | 9946 | -0.316 | -0.3657 | Yes |
| 75 | ARHGEF26 | Rho guanine nucleotide exchange factor 26 [Source:HGNC Symbol;Acc:HGNC:24490] | 9979 | -0.319 | -0.3594 | Yes |
| 76 | BMP6 | bone morphogenetic protein 6 [Source:HGNC Symbol;Acc:HGNC:1073] | 10021 | -0.323 | -0.3537 | Yes |
| 77 | NR2F2 | nuclear receptor subfamily 2 group F member 2 [Source:HGNC Symbol;Acc:HGNC:7976] | 10129 | -0.334 | -0.3528 | Yes |
| 78 | STARD13 | StAR related lipid transfer domain containing 13 [Source:HGNC Symbol;Acc:HGNC:19164] | 10183 | -0.339 | -0.3476 | Yes |
| 79 | TJP3 | tight junction protein 3 [Source:HGNC Symbol;Acc:HGNC:11829] | 10260 | -0.347 | -0.3440 | Yes |
| 80 | PECAM1 | platelet and endothelial cell adhesion molecule 1 [Source:HGNC Symbol;Acc:HGNC:8823] | 10304 | -0.351 | -0.3377 | Yes |
| 81 | FSTL1 | follistatin like 1 [Source:HGNC Symbol;Acc:HGNC:3972] | 10537 | -0.377 | -0.3454 | Yes |
| 82 | ROCK2 | Rho associated coiled-coil containing protein kinase 2 [Source:HGNC Symbol;Acc:HGNC:10252] | 10648 | -0.388 | -0.3433 | Yes |
| 83 | SOX17 | SRY-box transcription factor 17 [Source:HGNC Symbol;Acc:HGNC:18122] | 10843 | -0.410 | -0.3472 | Yes |
| 84 | JAG1 | jagged canonical Notch ligand 1 [Source:HGNC Symbol;Acc:HGNC:6188] | 10977 | -0.427 | -0.3458 | Yes |
| 85 | ACVR2B | activin A receptor type 2B [Source:HGNC Symbol;Acc:HGNC:174] | 11011 | -0.431 | -0.3364 | Yes |
| 86 | RAPGEF2 | Rap guanine nucleotide exchange factor 2 [Source:HGNC Symbol;Acc:HGNC:16854] | 11041 | -0.435 | -0.3267 | Yes |
| 87 | BMPR2 | bone morphogenetic protein receptor type 2 [Source:HGNC Symbol;Acc:HGNC:1078] | 11174 | -0.454 | -0.3245 | Yes |
| 88 | S1PR1 | sphingosine-1-phosphate receptor 1 [Source:HGNC Symbol;Acc:HGNC:3165] | 11230 | -0.461 | -0.3161 | Yes |
| 89 | PPP1R12A | protein phosphatase 1 regulatory subunit 12A [Source:HGNC Symbol;Acc:HGNC:7618] | 11259 | -0.465 | -0.3054 | Yes |
| 90 | BTG1 | BTG anti-proliferation factor 1 [Source:HGNC Symbol;Acc:HGNC:1130] | 11267 | -0.466 | -0.2931 | Yes |
| 91 | RAPGEF3 | Rap guanine nucleotide exchange factor 3 [Source:HGNC Symbol;Acc:HGNC:16629] | 11380 | -0.483 | -0.2885 | Yes |
| 92 | NOTCH4 | notch receptor 4 [Source:HGNC Symbol;Acc:HGNC:7884] | 11441 | -0.495 | -0.2796 | Yes |
| 93 | KDR | kinase insert domain receptor [Source:HGNC Symbol;Acc:HGNC:6307] | 11454 | -0.496 | -0.2668 | Yes |
| 94 | ITGAX | integrin subunit alpha X [Source:HGNC Symbol;Acc:HGNC:6152] | 11526 | -0.505 | -0.2584 | Yes |
| 95 | KDM6B | lysine demethylase 6B [Source:HGNC Symbol;Acc:HGNC:29012] | 11835 | -0.556 | -0.2672 | Yes |
| 96 | ROCK1 | Rho associated coiled-coil containing protein kinase 1 [Source:HGNC Symbol;Acc:HGNC:10251] | 12046 | -0.605 | -0.2669 | Yes |
| 97 | EDNRB | endothelin receptor type B [Source:HGNC Symbol;Acc:HGNC:3180] | 12075 | -0.613 | -0.2522 | Yes |
| 98 | GJA5 | gap junction protein alpha 5 [Source:HGNC Symbol;Acc:HGNC:4279] | 12095 | -0.619 | -0.2366 | Yes |
| 99 | IL1B | interleukin 1 beta [Source:HGNC Symbol;Acc:HGNC:5992] | 12105 | -0.622 | -0.2201 | Yes |
| 100 | FASN | fatty acid synthase [Source:HGNC Symbol;Acc:HGNC:3594] | 12194 | -0.643 | -0.2092 | Yes |
| 101 | FOXC2 | forkhead box C2 [Source:HGNC Symbol;Acc:HGNC:3801] | 12218 | -0.650 | -0.1931 | Yes |
| 102 | ACVRL1 | activin A receptor like type 1 [Source:HGNC Symbol;Acc:HGNC:175] | 12242 | -0.656 | -0.1768 | Yes |
| 103 | NOTCH1 | notch receptor 1 [Source:HGNC Symbol;Acc:HGNC:7881] | 12555 | -0.776 | -0.1798 | Yes |
| 104 | CLDN3 | claudin 3 [Source:HGNC Symbol;Acc:HGNC:2045] | 12615 | -0.807 | -0.1621 | Yes |
| 105 | STC1 | stanniocalcin 1 [Source:HGNC Symbol;Acc:HGNC:11373] | 12622 | -0.809 | -0.1402 | Yes |
| 106 | VEGFA | vascular endothelial growth factor A [Source:HGNC Symbol;Acc:HGNC:12680] | 12629 | -0.813 | -0.1183 | Yes |
| 107 | ROBO4 | roundabout guidance receptor 4 [Source:HGNC Symbol;Acc:HGNC:17985] | 12647 | -0.824 | -0.0969 | Yes |
| 108 | RHOB | ras homolog family member B [Source:HGNC Symbol;Acc:HGNC:668] | 12713 | -0.878 | -0.0777 | Yes |
| 109 | RAPGEF1 | Rap guanine nucleotide exchange factor 1 [Source:HGNC Symbol;Acc:HGNC:4568] | 12860 | -1.098 | -0.0589 | Yes |
| 110 | CTNNB1 | catenin beta 1 [Source:HGNC Symbol;Acc:HGNC:2514] | 12866 | -1.119 | -0.0284 | Yes |
| 111 | APOLD1 | apolipoprotein L domain containing 1 [Source:HGNC Symbol;Acc:HGNC:25268] | 12867 | -1.121 | 0.0026 | Yes |
Table: GSEA details [plain text format]

  

Fig 2: GOBP\_ENDOTHELIUM\_DEVELOPMENT      
 Blue-Pink O' Gram in the Space of the Analyzed GeneSet

  

Fig 3: GOBP\_ENDOTHELIUM\_DEVELOPMENT: Random ES distribution      
 Gene set null distribution of ES for **GOBP\_ENDOTHELIUM\_DEVELOPMENT**
